# Beyond the Middle Ear: A Thorough Review of Cholesteatoma in the Nasal Cavity and Paranasal Sinuses

**DOI:** 10.3390/diagnostics15121461

**Published:** 2025-06-08

**Authors:** Michail Athanasopoulos, Pinelopi Samara, Stylianos Mastronikolis, Sofianiki Mastronikoli, Gerasimos Danielides, Spyridon Lygeros

**Affiliations:** 1 Department of Otolaryngology, University Hospital of Patras, 26504 Patras, Greece; 3idani23@gmail.com (G.D.); slygeros@upatras.gr (S.L.); 2 Children’s Oncology Hospital Marianna V. Vardinoyannis-ELPIDA, 11527 Athens, Greece; psamara@biol.uoa.gr; 3 Department of Ophthalmology, Medical School, University of Patras, 26504 Patras, Greece; mastronikst@gmail.com; 4 Brighton and Sussex Medical School, Brighton BN1 9PX, UK; smastronikoli@gmail.com

**Keywords:** cholesteatoma, keratoma, nasal cholesteatoma, paranasal sinus cholesteatoma, sinus cholesteatoma

## Abstract

**Background:** Cholesteatoma, characterized by the abnormal growth of keratinizing squamous epithelium in ectopic locations, most commonly arises in the middle ear. Its occurrence in the sinonasal tract is rare and presents significant diagnostic and management challenges. These lesions can lead to severe complications like bone erosion, intracranial involvement, and orbital spread. This narrative review aims to summarize the current knowledge on cholesteatomas in these regions, focusing on epidemiology, pathophysiology, diagnosis, and treatment. **Methods:** A comprehensive review of the English literature was conducted, focusing on reported cases of cholesteatomas in the nasal cavity and paranasal sinuses. This review examines key aspects, including epidemiological data, imaging findings, surgical strategies, and postoperative outcomes. The role of diagnostic tools, particularly computed tomography and diffusion-weighted magnetic resonance imaging, in distinguishing cholesteatomas from other sinonasal lesions is also discussed. **Results:** As of March 2025, 51 cases of paranasal sinus cholesteatoma were reported. The frontal sinus is the most commonly affected site, followed by the maxillary, ethmoid, and sphenoid sinuses. Diagnosis is often delayed due to nonspecific symptoms, such as nasal congestion and recurrent infections. Surgical excision is the primary treatment, with endoscopic techniques being favored for their minimally invasive nature. Recurrence remains a major concern, and although very rare, cases of squamous cell carcinoma have also been observed in association with cholesteatoma. **Conclusions:** Nasal and paranasal sinus cholesteatomas require early recognition and intervention to prevent complications. Advances in imaging and surgery have improved outcomes; however, further research is needed to refine therapies and understand disease mechanisms.

## 1. Introduction

Cholesteatomas are expansile, keratinizing lesions most frequently encountered in the middle ear, but their occurrence in the nasal cavity and paranasal sinuses remains rare and poorly understood [1]. Despite their benign histology, these entities can exhibit locally aggressive behavior, leading to bone erosion, chronic infection, and even life-threatening complications. In the sinonasal tract, cholesteatomas pose significant diagnostic and therapeutic challenges due to their nonspecific clinical presentation and potential to mimic more common inflammatory or neoplastic processes [2].

The historical recognition of cholesteatoma in the nasal cavity and paranasal sinuses dates back to the early 20th century. Haeggström’s 1916 report describing a cholesteatoma of the frontal sinus represents one of the earliest documented cases in the literature [3], followed by Spencer’s 1930 account of a mass associated with orbital complications [4]. Despite these initial observations, sinonasal cholesteatomas remain significantly underexplored, particularly in comparison to their middle ear counterparts. Their rarity has contributed to a limited and fragmented body of literature, predominantly consisting of isolated case reports and small case series reports. As a result, substantial gaps persist in our understanding of their pathogenesis, as well as in the establishment of standardized diagnostic and therapeutic protocols.

Although chronic rhinosinusitis is a relatively common condition, the emergence of cholesteatoma within the nasal cavity or paranasal sinuses is exceptionally rare. This striking disparity highlights the distinctiveness of sinonasal cholesteatomas and underscores the need for heightened clinical suspicion, particularly in patients with atypical or refractory presentations. Furthermore, emerging reports of squamous cell carcinoma (SCC) arising within or adjacent to cholesteatomas raise important questions regarding the potential roles of chronic inflammation, epithelial instability, and microbial colonization in malignant transformation [5]. Recent technological advances have significantly enhanced diagnostic and therapeutic capabilities. High-resolution computed tomography (CT) and diffusion-weighted magnetic resonance imaging (MRI) facilitate the early and precise identification of these lesions. Additionally, the introduction and refinement of endoscopic sinus surgery have enabled more targeted and minimally invasive management, representing a notable advancement in the care of patients with sinonasal cholesteatomas.

Given the rarity and heterogeneity of sinonasal cholesteatomas, this review adopts a narrative format rather than a systematic approach, allowing for a comprehensive exploration of the historical context, clinical variability, and emerging concepts. This review aims to synthesize the current clinical knowledge on nasal and paranasal sinus cholesteatomas, with a focus on pathogenesis, diagnostic pitfalls, treatment modalities, and potential for malignancy. By critically examining the reported cases and evolving trends, we hope to offer practical insights for clinicians managing this rare but potentially serious condition.

## 2. Sources and Scope of the Literature

This narrative review synthesizes information from a broad selection of published literature on cholesteatoma of the nasal cavity and paranasal sinuses. Relevant sources were identified primarily through searches in PubMed, Scopus, and Google Scholar, with a focus on clinical case descriptions, pathological analyses, and radiological findings. Particular attention was given to case reports and small case series, which represent the majority of available data due to the rarity of this condition. The search was limited to English-language publications available up until March 2025, prioritizing studies that documented primary cholesteatoma arising within the paranasal sinuses. Studies in which the lesion extended into these regions from adjacent anatomical sites were excluded, as were studies lacking scientific rigor or sufficient methodological detail. The search terms included combinations such as “sinus cholesteatoma”, “nasal cholesteatoma”, “paranasal sinus cholesteatoma”, “frontal sinus cholesteatoma”, and “keratoma”, among others.

This is not a systematic review, and no formal review protocol was followed. Instead, a narrative approach was used to allow flexibility in discussing diverse sources and to contextualize them within the historical and clinical landscape of sinonasal cholesteatoma. This method was selected due to the limited number of studies and the heterogeneous nature of the available data. Our aim is to synthesize key findings while highlighting diagnostic and therapeutic developments, as well as gaps in the current understanding that merit further investigation.

## 3. Results

A survey of international English-language studies yielded 51 documented cases of cholesteatoma affecting the nasal cavity and paranasal sinuses as of March 2025. An overview of their anatomical distribution, clinical presentation, diagnosis, pathogenesis, and treatment outcomes is provided below.

### 3.1. Anatomical Distribution and Clinical Outcomes

**Figure 1:**
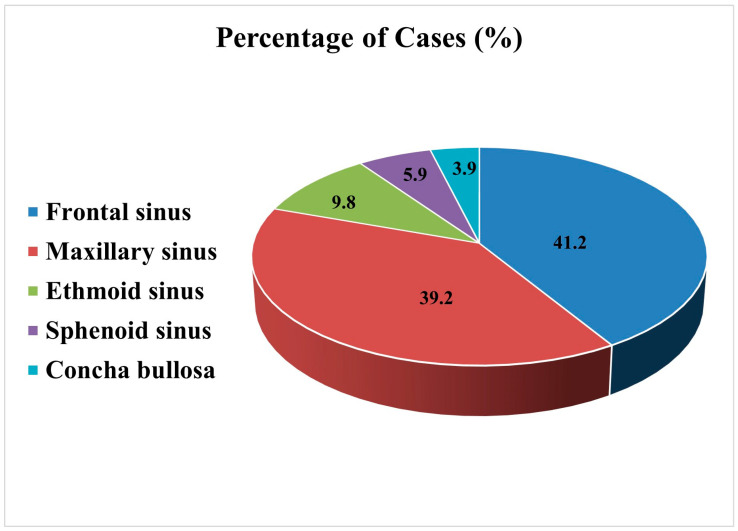
Anatomical distribution of cholesteatoma cases across the nasal and paranasal sinus sites. The percentages reflect the proportion of cases reported in the reviewed literature.

The disease exhibits a striking anatomical distribution, with the frontal sinus being the most commonly affected (41.2%), followed closely by the maxillary sinus (39.2%). Less frequent sites included the ethmoid sinus (9.8%), sphenoid sinus (5.9%), and concha bullosa (3.9%) (Figure 1). This distribution likely reflects variations in the sinus anatomy, drainage patterns, and susceptibility to trauma or chronic inflammation. The tendency for involvement of the frontal and maxillary sinuses may be linked to their complex ostial architecture and proximity to external or surgical insults, emphasizing the importance of anatomical context in disease pathogenesis and management. The following subsections present a descriptive, case-based overview organized by anatomical location, drawing on individual reports to highlight the observed clinical trends.

#### 3.1.1. Frontal Sinus

Cholesteatoma was most commonly found in the frontal sinus (21 cases) [2,4,6,7,8,9,10,11,12,13,14,15,16,17,18,19,20,21], with a male predominance (14 men and seven women). The patients ranged in age from 23 to 80 years, with several having relevant medical histories, such as prior sinus surgeries (three cases) and trauma (five cases). In this group, four cases progressed to SCC [4,8,11,13], and four experienced recurrences [7,10,15,18]. Tragically, three patients died due to disease-related complications, highlighting the severity of untreated cholesteatomas. These findings emphasize the aggressive nature of frontal sinus cholesteatomas and the importance of long-term follow-up, given their potential for recurrence and malignant transformation (Table 1).

#### 3.1.2. Maxillary Sinus

A total of 20 cases were found in the maxillary sinus, involving 11 men and nine women, with ages ranging from 1.5 to 76 years [5,22,23,24,25,26,27,28,29,30,31,32,33,34,35,36,37,38,39]. Relevant medical histories included prior nasal or sinus surgeries (three cases), tooth extractions (two cases), and surgery for congenital cleft lip and palate (one case). One patient died from vascular disease [37], and another developed SCC [5], illustrating the potential for malignancy in these cases. The involvement of previous dental and surgical interventions in some cases raises questions about the possible iatrogenic contributions to cholesteatoma formation (Table 2).

#### 3.1.3. Ethmoid Sinus

Cholesteatoma was identified in five cases within the ethmoid sinus (two men and three women), aged between 16 and 76 years [2,12,40,41,42]. Notably, one patient had undergone transsphenoidal pituitary surgery, while another had a history of nasal surgery (Table 3). These findings reflect the diverse clinical presentation of cholesteatomas. However, the small number of reported cases limits definitive conclusions regarding the risk factors and optimal management strategies.

#### 3.1.4. Sphenoid Sinus

Three cases of sphenoid sinus cholesteatoma have been documented, involving two men and one woman aged 25, 36, and 82 years, respectively [43,44,45]. None of the patients had a history of trauma or prior surgery in the region. All cases were surgically managed using a transsphenoidal approach, which is considered the safest approach for this area (Table 4). Unfortunately, one 82-year-old patient developed skull base osteomyelitis, a life-threatening complication that leads to severe neurological and infectious outcomes [45]. This case underscores the critical need for early diagnosis and appropriate surgical intervention to prevent such complications. Given the proximity of the sphenoid sinus to vital neurovascular structures, even small lesions may have devastating consequences.

#### 3.1.5. Concha Bullosa

Two cases were identified in the concha bullosa, affecting women aged 24 and 81 years [2,46]. Neither patient had a history of surgery or trauma in the relevant area. Endoscopic sinus surgery was performed, with no recurrence observed during follow-up, highlighting the success of minimally invasive techniques in managing cholesteatoma (Table 4). While rare, the occurrence of cholesteatoma in concha bullosa raises intriguing questions about the role of anatomical variations in its pathogenesis.

### 3.2. Gender and Age Patterns: Who Gets Affected and When?

Among the reviewed cases, 60.8% involved male patients, suggesting possible male predominance. This disparity may reflect anatomical or hormonal differences and varying exposure to risk factors, such as trauma or surgical intervention. Patients span a wide age range from infancy (1.5 years) to elderly adults (82 years), with notable differences between sexes [2,3,4,5,6,7,8,9,10,11,12,13,14,15,16,17,18,19,20,21,22,23,24,25,26,27,28,29,30,31,32,33,34,35,36,37,38,39,40,41,42,43,44,45,46]. Among males, cases are reported between the ages of 10 and 76 years, with a higher incidence observed in middle-aged and older individuals than in younger individuals. Interestingly, in females, a broader age distribution is evident, including cases in very young patients, even as early as infancy (1.5 years old). The presence of cases in children and adolescents suggests the possibility of alternative pathogenic mechanisms beyond acquired factors, such as trauma or sinonasal surgery. In females, earlier onset may indicate congenital anomalies or disruptions in epithelial migration during development [29]. These observations raise the hypothesis of potential sex-specific predispositions to disease onset and pathophysiology. The higher median age observed in men may reflect the cumulative impact of chronic inflammatory processes, repeated mechanical microtrauma, or progressive obstruction of the natural drainage pathways of the paranasal sinuses. These chronic exposures may contribute to the delayed manifestation of cholesteatoma in this subgroup of patients.

### 3.3. Localization and Symptoms: Anatomy Drives the Agenda

The clinical presentation of cholesteatoma in the nasal cavity and paranasal sinuses is highly variable and largely dependent on the specific sinus involved. The disease often progresses insidiously, remaining asymptomatic until significant keratin accumulation and tissue destruction result in localized or systemic symptoms. In the frontal sinus, patients usually present with frontal headache, proptosis, confusion, and, in more severe cases, fever or orbital swelling, which may suggest an intracranial extension [12,14]. Ethmoid sinus involvement is typically associated with medial canthal swelling [12], retro-orbital pain [40], and symptoms resembling allergic rhinitis [2]; orbital invasion in this region can also lead to proptosis and eyelid edema. When the maxillary sinus is affected, individuals may experience tooth pain, reduced vision, epiphora, or facial swelling due to the close anatomical relationship between the sinus, orbit, and dental roots [22,39]. Involvement of the sphenoid sinus often results in deep-seated headaches, diplopia, or cranial nerve palsy, given its proximity to the optic nerve and cavernous sinus [43,44,45]. Lastly, cholesteatoma arising within a concha bullosa may manifest as medial nasal swelling and purulent nasal discharge, with favorable outcomes frequently observed following endoscopic surgical resection [2,46].

### 3.4. Pathogenesis: Inflammation or Entrapment?

The development of cholesteatoma in the nasal cavity and paranasal sinuses reflects a multifaceted pathophysiological process involving both congenital and acquired factors [31]. Although rare, its predilection for the frontal and maxillary sinuses highlights the role of local anatomy and environmental stressors -such as trauma, prior surgery, chronic infection, and impaired drainage- in driving disease onset and progression [11,13]. In acquired cases, epithelial entrapment plays a central role [47]. Repeated episodes of inflammation or mechanical disruption from surgical manipulation can lead to the displacement of the squamous epithelium into areas where it does not naturally belong [12,14]. Once sequestered, the epithelium may undergo unchecked proliferation, supported by a pro-inflammatory microenvironment. This is particularly evident in the maxillary sinus, where chronic infection and disrupted mucociliary function foster a cycle of persistent irritation and keratin debris accumulation [24]. In contrast, in the frontal sinus, surgical trauma, often from prior sinus interventions, appears to be a more dominant initiating factor, correlating with the clinical histories of many reported cases. As cholesteatomas expand, they behave aggressively despite their benign histology. The accumulation of keratinous material exerts pressure on adjacent tissues, and associated inflammation accelerates local bone resorption. This destructive potential can result in complications such as osteomyelitis, orbital wall erosion, and cerebrospinal fluid leakage through the skull base. Moreover, chronic inflammatory insults may induce epithelial metaplasia or dysplasia, raising questions about potential neoplastic transformation in long-standing cases.

### 3.5. Cholesteatoma or Neoplasm? The Diagnostic Gray Zone

Although cholesteatoma is histologically benign, a small number of cases have raised concerns regarding its potential association with SCC. To date, five cases of SCC arising in the context of paranasal cholesteatoma have been documented: four in the frontal sinus [4,8,11,13] and one in the maxillary sinus [5]. This rare but serious occurrence has prompted speculation regarding the possible mechanisms underlying malignant transformation [5].

Chronic inflammation, persistent microbial colonization (notably by *Pseudomonas aeruginosa* or human papillomavirus [HPV]), and repeated epithelial irritation may contribute to a microenvironment conducive to dysplasia or neoplastic changes [48]. It remains uncertain whether SCC, in these cases, represents the true malignant transformation of pre-existing cholesteatoma or arises independently within a milieu of chronic inflammation and tissue destruction. Histologically and radiographically, distinguishing between aggressive cholesteatoma and early-stage carcinoma can be challenging, particularly in extensively inflamed or remodeled sinuses. This diagnostic ambiguity underscores the need for a high clinical suspicion, thorough histopathological evaluation, and long-term follow-up in atypical or refractory cases.

### 3.6. Challenging the Diagnosis: Seeing Through the Mucosal Fog

Diagnosing cholesteatoma in the paranasal sinuses is inherently challenging due to its rarity, insidious onset, and nonspecific clinical presentation. The absence of pathognomonic symptoms and radiological overlap with other inflammatory or neoplastic conditions necessitate a comprehensive and vigilant diagnostic strategy. Early recognition and accurate differentiation from other sinonasal diseases are essential, particularly in light of potential complications such as bone erosion and extension into adjacent critical structures, including the orbit and anterior skull base. Cholesteatomas in this region often behave as expansile lesions that gradually infiltrate multiple sinuses, frequently extending beyond their site of origin. This can result in widespread disease involving the maxillary, ethmoid, sphenoid, and frontal sinuses, often accompanied by bone destruction and remodeling.

Nasal endoscopy is the first-line diagnostic tool, offering direct, real-time visualization of the nasal cavity and middle meatus. A meticulous endoscopic examination may reveal hallmark features, such as a white, grayish, or yellowish friable mass composed of keratinized squamous epithelium [31]. Depending on the chronicity of the disease, the lesion may appear smooth or irregular. Additional findings may include mucosal swelling, hyperemia, and obstruction of the sinus ostia, which can predispose patients to mucus retention and secondary infections. In some cases, physical examination or anterior rhinoscopy may detect the lesion, particularly when it extends into the nasal cavity, as in the case of a 10-year-old boy who presented with total nasal obstruction and a whitish mass occupying the entire left nasal passage [32]. However, for lesions located deeper within the paranasal sinuses, which may not be fully visible through endoscopic examination of the nasal cavity, imaging is essential for evaluating the extent of the lesion, assessing any damage to surrounding structures and determining its spread.

### 3.7. Imaging Matters: From Shadows to Structures

The journey of diagnosing cholesteatoma in the nasal cavity and paranasal sinuses has evolved dramatically with advancements in imaging technologies. Before the 1980s, conventional radiography was the primary diagnostic tool [7,25]. These radiographs are useful for detecting gross infections, inflammation, and anatomical deviations; however, their limitations quickly become apparent. X-rays lack the resolution necessary to visualize delicate sinus structures, detect small lesions, or reliably assess bone integrity. The inability to distinguish between benign inflammatory changes and malignant growths often leaves clinicians with diagnostic ambiguity.

The emergence of CT and MRI has transformed this landscape. These modalities have ushered in a new era of structural clarity, offering precise anatomical details and functional insights. CT scans have become indispensable for evaluating cholesteatoma [30,41]. They effectively reveal bone erosion, sinus opacification, and obstruction of the natural drainage pathways [2]. Typical CT findings include a soft-tissue mass with well-demarcated bone destruction, indicative of a cholesteatoma. In chronic cases, subtle changes, such as bone remodeling, thickening, or rarefaction, may also be evident, reflecting the pressure and persistence of the lesion over time [32]. MRI complements CT by providing superior soft-tissue contrast and more nuanced lesion characterization. Cholesteatomas generally exhibit low signal intensity on T1-weighted images and high intensity on T2-weighted sequences, consistent with their keratin and cholesterol-rich contents. MRI is especially valuable in evaluating suspected complications, such as orbital or intracranial extension, and in differentiating cholesteatoma from neoplastic or other inflammatory entities that may clinically and radiologically mimic it [19].

Together, CT and MRI form the cornerstone of the modern diagnostic work-up for paranasal cholesteatomas. Their integration not only enhances diagnostic confidence but also informs surgical planning and long-term management of the disease. In a condition where clinical signs may be vague and endoscopic visibility is limited, imaging bridges the gap by transforming shadows into structures and uncertainty into clarity.

### 3.8. Surgical Treatment of Cholesteatoma in the Paranasal Sinuses

Managing cholesteatoma in the nasal cavity and paranasal sinuses presents unique surgical challenges, which are shaped by the aggressive behavior of the lesion and its proximity to critical anatomical structures. Historically, the goal of treatment has been complete excision to prevent recurrence and limit complications, such as bone erosion, intracranial extension, and orbital invasion. However, the path to achieving this goal has evolved considerably over the years [15,49].

#### 3.8.1. From Open Surgery to Endoscopy: A Paradigm Shift

In the pre-imaging and pre-endoscopy era, open surgical approaches, such as trepanation, osteoplastic flap techniques, lateral rhinotomy, and the modified Krönlein approach, were the cornerstone of treatment, providing the extensive exposure required to visualize and excise disease from complex sinus recesses. The supraorbital incision, for example, enables direct access to the frontal sinus in cases at risk for optic nerve compression [10], while lateral rhinotomy facilitates the removal of lesions in the ethmoid and maxillary sinuses, particularly in advanced or anatomically distorted presentations [12]. Although effective in affording wide exposure, these techniques have significant drawbacks. Incomplete excision frequently leads to high recurrence rates, residual cavities predisposed to mucocele formation and chronic osteitis, and serious complications, including meningeal inflammation, intracranial extension, and, in rare instances, malignant transformation [13,16]. Aesthetic and functional sequelae, such as visible facial scars, bone defects, and prolonged recovery times, underscore the need for less invasive alternatives.

The advent of endoscopic sinus surgery has revolutionized the management of sinus disease by offering a minimally invasive route that preserves normal anatomy and function. Through angled endoscopes and powered instrumentation, surgeons can now access and clear disease from all sinus compartments, including previously hard-to-reach areas [10,15,49].

#### 3.8.2. Endoscopic Surgery: Panacea or Partial Fix?

Endoscopic techniques have truly transformed the treatment landscape for cholesteatoma in the nasal cavity and paranasal sinuses. Once managed primarily through invasive open techniques, these lesions can now often be addressed with precision and minimal disruption, thanks to high-resolution endoscopes and advanced surgical navigation systems. [39] This shift toward less invasive methods has redefined surgical priorities, emphasizing enhanced visualization, preservation of function, and reduced morbidity [15,50]. Endoscopic approaches offer several clear advantages. Unlike traditional open surgeries that require large facial or cranial incisions, endoscopic techniques are performed entirely through the nasal passages, eliminating cosmetic concerns and significantly shortening the healing phase. Enhanced intraoperative visualization enables thorough dissection, while navigation systems help minimize the risk of injury to adjacent neurovascular structures. Additionally, by restoring natural sinus drainage pathways, endoscopic surgery reduces the chances of mucocele formation and recurrent infections [15,50].

The versatility of endoscopic techniques has been demonstrated in nearly all sinus regions. In the frontal sinus, the endoscopic modified Lothrop procedure (Draf III) is commonly employed for localized cholesteatoma, offering direct access to the frontal recess and improved postoperative drainage [17,19]. For more extensive disease or anterior skull base involvement, a hybrid approach incorporating an osteoplastic flap may be necessary [21]. The ethmoid sinuses, frequently affected in cases presenting with orbital symptoms such as proptosis or periorbital swelling, are typically addressed with endoscopic ethmoidectomy [2]. In the maxillary sinus, where the lesion may erode bone or compress adjacent structures, endoscopic middle meatal antrostomy provides effective drainage and functional preservation [5,35]. However, in cases marked by significant bone destruction or recurrence, the traditional Caldwell-Luc approach may still be warranted [24]. Deep-seated sphenoid sinus cholesteatomas, given their proximity to the optic nerve and cavernous sinus, are optimally accessed via transnasal transsphenoidal endoscopic techniques, minimizing the need for external craniotomy [43,44,45].

Despite these advantages, endoscopy is not a universal diagnostic tool. In cases of aggressive, recurrent, or extensively spreading cholesteatomas, endoscopic access alone may be insufficient. A limited surgical corridor can hinder the complete excision of deeply embedded or posteriorly located tissues. In such scenarios, open or combined approaches remain indispensable, offering broader exposure and facilitating radical clearance. Thus, while endoscopic sinus surgery represents a significant advancement in the management of sinonasal cholesteatoma, it is not a panacea. Its success lies in careful patient selection, precise anatomical understanding, and when needed, the thoughtful integration of open techniques. As surgical tools and imaging continue to improve, the balance between endoscopy and traditional approaches will evolve further to optimize long-term disease control.

#### 3.8.3. Complications, Monitoring, and the Road Ahead

Despite significant advances in surgical techniques and postoperative care, managing cholesteatoma in the nasal cavity and paranasal sinuses remains challenging. Success depends not only on technical precision during surgery but also on the diligence of long-term monitoring. Both endoscopic and open procedures carry the risk of complications, the most common of which is disease recurrence, often attributed to residual keratinizing epithelium or incomplete excision [20]. In more severe cases, erosion of adjacent structures, such as the skull base, can lead to life-threatening complications, including frontal lobe abscesses or meningoencephalitis [16,18]. Chronic inflammation, especially when compounded by repeated infections or incomplete surgical clearance, may even set the stage for rare but serious outcomes like malignant transformation [5,8,11,13].

In light of these risks, diligent postoperative monitoring is not merely advisable; it is essential. Regular nasal endoscopy allows direct visualization of the surgical site, enabling the detection of early signs of recurrence, persistent inflammation, or secondary infections. Endoscopic surveillance is particularly critical in anatomically challenging areas, such as the posterior frontal recess and sphenoid sinus, where residual disease can easily go unnoticed during surgery [15,20]. Radiological follow-up plays a complementary role in this context. Non-contrast CT scans remain the gold standard for assessing bony changes and sinus architecture, while MRI offers superior soft tissue contrast, allowing differentiation of postoperative changes from residual or recurrent cholesteatoma. The ability to monitor complications such as bone erosion, mucoceles, or even intracranial extension further highlights the indispensable role of imaging in long-term care. Pharmacological therapy continues to support surgical management, particularly during the postoperative phase. Antibiotics are frequently administered to address secondary bacterial infections, while corticosteroids may help reduce inflammation and improve mucosal healing. In certain cases, antifungal treatment is necessary, particularly in immunocompromised patients or those with fungal colonization [10,15].

Eventually, the long-term prognosis hinges on consistent and individualized follow-up. Patients with incomplete excision or aggressive disease phenotypes require close surveillance to mitigate the risk of recurrence and monitor potential malignant changes. The frequency of follow-up should be tailored to the clinical scenario, but vigilance must be sustained over years rather than over months. Prompt recognition of new or worsening symptoms, such as unexplained facial pain, vision changes, or persistent discharge, can be critical, serving as early warning signs of recurrence or transformation [15].

## 4. Discussion

This review synthesizes clinical, radiological, and pathological data from 51 reported cases of nasal and paranasal sinus cholesteatoma, revealing key insights into its anatomical distribution, clinical presentation, and associated therapeutic challenges. The frontal and maxillary sinuses emerged as the most frequently affected sites, a trend likely linked to their anatomical susceptibility to obstruction, infection, and injuries. The frontal sinus, with its narrow drainage pathway and frequent exposure to surgical manipulation or chronic inflammation, appears to be particularly predisposed. Similarly, the maxillary sinus, due to its proximity to both the nasal and oral cavities, may serve as a locus for infection and iatrogenic injury, further predisposing it to cholesteatoma formation. These observations underscore the necessity of site-specific considerations in diagnosis and treatment planning.

Clinically, paranasal cholesteatomas often present with nonspecific symptoms, such as nasal obstruction, headache, and foul-smelling nasal discharge, making early recognition difficult. However, as the disease progresses, more severe and potentially life-threatening complications can arise, including skull base osteomyelitis, orbital invasion, and intracranial spread of the disease. Several cases reviewed resulted in significant morbidity and, in rare instances, mortality, underscoring the importance of maintaining a high index of suspicion in patients with recurrent or atypical sinus disease, particularly when radiologic findings are incongruent with common inflammatory pathologies.

In the differential diagnosis of paranasal cholesteatomas, several rare but clinically significant entities must be considered. These include congenital or developmental lesions, such as teratomas, dermoid cysts, and epidermoid cysts, which may present with overlapping radiologic or clinical features. Additionally, acquired conditions such as mucoceles, inverted papillomas, and various benign or malignant tumors can mimic cholesteatomas in terms of appearance and behavior. An accurate diagnosis requires a comprehensive approach that integrates clinical assessment, advanced imaging, and histopathological evaluation. Although not designed to specifically identify cholesteatoma, validated symptom-scoring instruments encompass both nasal and otologic domains and may assist in detecting atypical or overlapping symptom profiles. Their application in routine assessment could support earlier diagnostic consideration of rare entities when standard inflammatory pathologies do not fully account for the clinical picture.

Demographic analysis revealed a male predominance among reported cases, with affected individuals ranging in age from 1.5 to 82 years old. Interestingly, pediatric cases—particularly in females—raise the possibility of congenital or developmental factors in pathogenesis, complementing the more commonly implicated acquired causes such as chronic infection, surgery, or trauma. This demographic pattern suggests a multifactorial origin shaped by both environmental exposure and intrinsic anatomical or immunological vulnerabilities.

A particularly concerning aspect of nasal and paranasal cholesteatomas is their rare but reported potential for malignant transformation. In the literature, five cases of SCC arising within or adjacent to cholesteatoma lesions—most commonly in the frontal and maxillary sinuses—have been described. While these reports raise important clinical questions, the limited number of cases precludes any definitive conclusions regarding causality or statistical correlation. Nevertheless, it has been hypothesized that persistent inflammation, epithelial dysplasia, and chronic microbial colonization (e.g., *Pseudomonas aeruginosa* or HPV) may contribute to a microenvironment favorable for neoplastic change. Although the incidence appears low, these rare occurrences underscore the need for vigilance and further research in long-term follow-ups. Given the chronic inflammatory milieu characteristic of sinonasal cholesteatomas, the widespread use of intranasal corticosteroids warrants further consideration. While these agents effectively reduce inflammation, prolonged topical use may alter epithelial integrity and local immune responses, potentially promoting epithelial irritation or dysregulation. Although current evidence is limited, investigating the effects of commonly used intranasal medications on the pathogenesis and progression of these lesions represents a valuable direction for future research.

Surgical excision remains the cornerstone of the treatment. With the advent of endoscopic sinus surgery, the ability to achieve precise lesion removal while minimizing morbidity has improved significantly. However, recurrence remains a major concern, typically due to incomplete excision, especially in anatomically complex or poorly visualized regions. This reinforces the need for meticulous intraoperative techniques and sustained postoperative surveillance. High-resolution imaging (CT for bony details and MRI for soft tissue involvement) is essential for evaluating residual disease and guiding any necessary reintervention. Pharmacological management, including the use of antibiotics for secondary infections, corticosteroids for inflammation, and antifungals when indicated, plays a supportive role but does not replace the need for definitive surgical treatment. An individualized multidisciplinary strategy that combines surgical precision with regular endoscopic monitoring and tailored medical therapy offers the best opportunity for durable disease control. Finally, the importance of vigilant long-term follow-up cannot be overestimated. Recurrence may present subtly or mimic other sinus disorders, and new or worsening symptoms—such as facial pain, proptosis, or changes in olfaction—should prompt immediate evaluation. Early detection and timely intervention are critical for preventing complications and improving long-term outcomes.

This narrative review has some limitations. The rarity of sinonasal cholesteatoma requires reliance on isolated case reports and small series, which may introduce publication bias and limit generalizability. While efforts were made to include a comprehensive range of sources, access limitations to certain full-text articles may have affected the overall completeness of the literature synthesis. Additionally, the exclusion of non-English publications and studies lacking methodological details may have further constrained the scope. Despite these limitations, this review offers a structured summary of the current knowledge and highlights areas requiring further investigation.

## 5. Conclusions

Although uncommon, cholesteatoma of the paranasal sinuses poses a significant clinical challenge due to its capacity for local destruction, recurrence, and, in rare cases, malignant transformation. Advances in imaging modalities and the adoption of endoscopic surgical techniques have markedly improved diagnostic accuracy and reduced treatment-related morbidity. Despite these developments, the complex pathophysiology, anatomical variability, and potential for delayed diagnosis continue to complicate management. Long-term postoperative surveillance remains critical for the early detection of recurrence and prevention of complications. Moving forward, a comprehensive and individualized approach combining precise surgical intervention, robust follow-up protocols, and adjunctive pharmacologic support will be essential for optimizing patient outcomes.

## 6. Future Directions

Future research should elucidate how chronic inflammation, microbial colonization, and key molecular pathways contribute to malignant transformation, especially to SCC. Identifying specific biomarkers and pathogenic mechanisms could facilitate earlier diagnosis and pave the way for tailored therapies. The incorporation of artificial intelligence and machine learning tools into diagnostic algorithms promises to enhance precision and anticipate recurrence risk. Moreover, given the rarity and heterogeneity of sinonasal cholesteatoma, establishing standardized diagnostic and therapeutic protocols is crucial for improving patient outcomes. This will require coordinated multidisciplinary efforts and the creation of collaborative registries or databases to enable comprehensive data sharing and collective analysis across centers. These strategies will aid in the development of evidence-based diagnostic pathways and treatment guidelines. Concurrently, the ongoing refinement of minimally invasive surgical approaches, augmented by next-generation navigation systems and real-time intraoperative imaging, will further decrease patient morbidity and optimize outcomes. By harnessing advances in molecular medicine and cutting-edge surgical technologies, we can fundamentally improve the management of this rare yet challenging disease.

## Figures and Tables

**Table 1:** Reported cases of frontal sinus cholesteatomas in the English literature.

Author (Year)	Gender/ Age	Primary Symptoms	Trauma History/ Surgery	Management	Malignancy or Intracranial Complication	Outcome
Spencer (1930) [4]	M/40	Exophthalmos, decreased vision	Cranial trauma	Trepanation method (Krönlein)	Exposure of the right frontal lobe, paraplegia	SCC, death 14 years later
Coates (1937) [6]	M/51	Purulent discharges, proptosis, orbital swelling	Cranial trauma	Orbital decompression, drainage of posterior orbital abscess, mass removal	Dura thickening due to chronic inflammation	No recurrence 12 months later
	F/27	Supraorbital edema, frontal headache	-	Sinusotomy via an external approach	Exposure of the dura mater, presence of granulation tissue, necrotic perforation of the anterior wall, subperiosteal abscess	The patient missed the follow-up 10 months later
Thacker (1950) [7]	M/26	Proptosis	Cranial trauma	External approach, Lynch-type incision	Mass in direct contact with the dura	Recurrence 5 years later
Osborn (1967) [8]	F/62	Pain, proptosis/edema	Sinus surgery	Excision via extracranial approach	SCC 5 years after the surgery	Loss of consciousness, disorientation, brain invasion, death
Calcaterra (1976) [9]	M/44	Proptosis	-	Not reported	Not reported	Not reported
Holt (1977) [10]	F/24	Exophthalmos, loss of vision	-	External approach, supraorbital incision	Severe bone destruction, intact dura	Recurrence 30 years later
Maniglia (1977) [11]	M/67	Frontal headache, nasal obstruction	-	Not reported	-	SCC
Campanella (1979) [12]	M/65	Proptosis, double vision	-	Left supraorbital approach with an osteoplastic flap via an eyebrow incision	-	No recurrence 10 years later
	M/46	Frontal headache	-	Approach to the right frontal sinus using an osteoplastic flap	-	Not reported
	M/36	Right eyelid edema	-	Exploration via an upper eyelid incision	Erosion of the right frontal bone, dura exposure	Death from a car accident 4 months later
Newman (1983) [13]	M/70	Blurred vision, proptosis	-	Approach to the right frontal sinus using an osteoplastic flap	Verrucous carcinoma, frontal lobe abscess	Death on the 7th postoperative day after abscess surgery
Hopp (1984) [14]	M/23	Frontal headache	Mild forehead trauma	Approach to the frontal sinus with an osteoplastic flap	-	No recurrence 12 months later
Hansen (2007) [15]	M/80	Frontal headache, tenderness on palpation, blurred vision, left eye proptosis	-	Sinus surgery, ethmoidectomy via an external approach	Erosion of the posterior wall of the frontal sinus, dura mater with a shiny, keratin-like appearance	Recurrence 10 months later
Hammami (2010) [16]	M/25	Upper eyelid edema, fever, altered consciousness, generalized seizures	-	Frontal sinus approach via an eyebrow incision after bony flap removal	Bacterial meningo-encephalitis	No recurrence 5 months later
Lai (2010) [17]	M/53	Rhinorrhea, postnasal drip, anosmia	-	Functional endoscopic sinus surgery (modified Lothrop procedure)	Extensive bone erosion, dura exposure	No recurrence 24 months later
Zoia (2018) [18]	M/32	Frontal headache, painful frontal swelling, fever, eyelid edema	-	Endoscopic sinus surgery	Frontal lobe abscess	Recurrence 6 months later, re-operation
Kurien (2019) [19]	F/27	Headache, forehead swelling	-	Endoscopic surgery of the nose and paranasal sinuses (Lothrop procedure) combined with an external approach via an incision along the hairline	Dura mater adherent to both the bone and the cholesteatoma at the sites of bone defects	No recurrence 6 months later
Tejani (2020) [20]	M/45	Forehead and eyelid swelling	Repeated head injuries during childhood	Endoscopic sinus surgery	Dura exposure	Mild keratin remnants in the frontal sinus 2.5 years later; irrigation/follow-up
Goncalves (2022) [21]	F/68	Headache, fever, right eyelid edema/ptosis, exophthalmos, conjunctival redness	Craniotomy, endoscopic approach for subcutaneous abscess of the frontal region	Draf III, external approach via an incision along the hairline	-	No recurrence 6 months later
Ahmed (2023) [2]	F/35	Frontal headache	External approach to the right frontal sinus via an eyebrow incision	Endoscopic sinus surgery, external trepanation	-	No recurrence 24 months later

**Table 2:** Reported cases of maxillary sinus cholesteatoma in the English literature.

Author (Year)	Gender/ Age	Primary Symptoms	Trauma History/ Surgery	Management	Malignancy or Intracranial Complication	Outcome
Mills (1958) [22]	F/65	Left facial pain/swelling, rhinorrhea, nasal obstruction	-	Opening of an oroantral fistula, surgical excision, antrostomy	-	Not reported
Pogorel (1965) [23]	M/36	Toothache, headache, swelling/pain of the left eye, diplopia, fever, following tooth extraction	-	Maxillary sinus antrostomy, Caldwell-Luc procedure	-	No recurrence 24 months later
Baxter (1966) [24]	M/20	Left facial swelling	Nasal surgery for polyp removal	Caldwell Luc procedure	-	Not reported
Das (1971) [25]	F/55	Facial pain, nasal obstruction	-	Caldwell Luc procedure	-	Not reported
Paaske (1984) [26]	M/76	Facial pain, intermittent purulent discharge from the left nostril	Facial trauma, tooth extractions in the upper jaw	Caldwell Luc procedure	-	Not reported
Sadoff (1989) [27]	F/55	Left facial swelling, nasal congestion	-	Nasal antrostomy	-	No recurrence 8 months later
Storper (1992) [28]	M/12	Left facial swelling, pain in the upper jaw/hard palate, nasal obstruction, clear nasal discharge, periodic generalized headaches	-	Caldwell Luc procedure	-	No recurrence 8 months later
Vaz (2000) (congenital) [29]	F/1.5	Right facial and intraoral swelling, snoring, epiphora	-	Inferior nasal antrostomy of the maxillary sinus	-	No recurrence, time interval not specified
Palacios (2005) [30]	M/57	Left facial swelling	-	Not reported	-	Not reported
Viswanatha (2007) [31]	F/18	Painless swelling of the left buccal region, nasal obstruction, intermittent headaches	-	Approach via an incision inside the mouth, beneath the lip	-	No recurrence 6 months later
Viswanatha (2011) [32]	M/10	Left nasal obstruction, intermittent foul-smelling nasal discharge	Cleft lip/palate repair	Endoscopic sinus surgery	-	No recurrence 6 months later
Chouhan (2011) [33]	F/47	Right nasal obstruction with discharge, facial swelling	-	Endoscopic sinus surgery	-	Not reported
Buric (2013) [34]	F/37	Mild, dull pain and a sensation of swelling in the upper jaw in the area of the left molars	-	Intraoral excision	-	No recurrence 6 months later
Sozansky (2015) [35]	M/72	Recurrent rhinosinusitis, nasal congestion, postnasal drip with colored mucous discharge	-	Endoscopic sinus surgery	-	No recurrence 13 years later
Jin (2016) [36]	F/34	Facial pain, toothache, recurrent purulent discharge from the left nostril	Endoscopic sinus surgery	Caldwell Luc procedure	-	No recurrence 12 months later
Bourchom (2017) [37]	F/74	Facial/hard palate swelling, nasal obstruction	-	Caldwell Luc procedure, middle nasal antrostomy	-	Death from vascular disease after surviving without recurrence for 18 months
Vakalapudi (2021) [38]	M/36	Toothache, facial swelling, pus drainage	Tooth extraction	Biopsy and further referral to a maxillofacial surgery clinic	-	Not reported
Zhen (2022) [39]	M/64	Nasal obstruction, postnasal discharge	Endoscopic sinus surgery	Endoscopic sinus surgery	-	No recurrence 32 months later
	M/31	Swelling of the right buccal region, obstruction of the right nasal cavity, toothache, epiphora	-	Endoscopic sinus surgery	-	No recurrence 12 months later
Kim (2024) [5]	M/40	Dull pain in the left orbital cavity, foul-smelling nasal discharge	-	Caldwell Luc procedure, Endoscopic sinus surgery	-	SCC, radio-chemotherapy, no recurrence 1 year later

**Table 3:** Reported cases of ethmoid sinus cholesteatomas in the English literature.

Author (Year)	Gender/ Age	Primary Symptoms	Trauma History/ Surgery	Management	Malignancy or Intracranial Complication	Outcome
Campanella (1979) [12]	M/48	Painless swelling on the medial side of the right eye	Nasal surgery	Lateral rhinotomy	-	No recurrence, follow-up duration not specified
Cingi (1991) [40]	F/16	Painful, progressive swelling of the right eye, proptosis	-	Lateral rhinotomy	-	No recurrence 26 months later
Barnett (1992) [41]	M/76	Transient episode of confusion/disorientation, frontal area protrusion, conductive hearing loss, anosmia	-	Craniotomy	Bilateral displacement of the frontal lobes, hyperplastic lesions of the skull, anterior compression of the ventricles, mild hypodense lesion in the white matter posterior to the mass	No new neurological deficits, with impressive reexpansion of the frontal hemispheres
Chandra (2006) [42]	F/22	Headache	Frontal trauma, motor vehicle accident, transsphenoidal hypophysectomy of a macroadenoma	Endoscopic sinus surgery, trepanation	-	No recurrence 14 months later
Ahmed (2023) [2]	F/17	Medial canthus swelling, headache, symptoms of allergic rhinosinusitis	-	Endoscopic sinus surgery	-	No recurrence 24 months later

**Table 4:** Reported cases of sphenoid sinus and concha bullosa cholesteatomas in the English literature.

Author (Year)	Anatomical Site	Gender/ Age	Primary Symptoms	Trauma History/ Surgery	Management	Malignancy or Intracranial Complication	Outcome
Ohta (1997) [43]	Sphenoid sinus	M/36	Diplopia, sixth cranial nerve paresis	-	Transsphenoidal approach	Extension toward the clivus and the brainstem	Not reported
Sani (2005) [44]		M/25	Headache, diplopia, reduced visual acuity, upper eyelid ptosis, third cranial nerve palsy	-	Transsphenoidal approach	Extension into the sella turcica presenting as pituitary apoplexy	Complete neurological recovery 3 months later
Kanjanawasee (2019) [45]		F/82	Chronic headache, fever	-	Transsphenoidal approach	Skull base osteomyelitis	Death
Cukurova (2010) [46]	Concha bullosa	F/81	Headache, diplopia, eye proptosis, nasal obstruction	-	Endoscopic sinus surgery	-	No recurrence 14 months later
Ahmed (2023) [2]		F/24	Nasal obstruction, external swelling beneath the left eye	-	Endoscopic sinus surgery	-	No recurrence 24 months later

## Data Availability

This review is based on the currently available literature.

## Abbreviations

SCC	Squamous cell carcinoma
CT	Computed Tomography
MRI	Magnetic resonance imaging
HPV	Human papilloma virus

## References

[1] Schürmann M., Goon P., Sudhoff H. (2022). Review of potential medical treatments for middle ear cholesteatoma. Cell Commun. Signal..

[2] Ahmed A.N.A., Elsharnouby M.M., Elbegermy M.M. (2023). Nasal sinuses cholesteatoma: Case series and review of the English literature. Eur. Arch. Oto-Rhino-Laryngol..

[3] Haeggström A.A. (1916). Ett funn av cholesteatoma i pannhulan. Hygiea.

[4] Spencer F.R. (1930). Primary cholesteatoma of the sinuses and orbit: Report of a case of many years’ duration followed by carcinoma and death. Arch. Otolaryngol. Head Neck Surg..

[5] Kim T., Whang C., You J., Park H., Geum S., Shin S., Ye M. (2024). Squamous cell carcinoma arising from a cholesteatoma of the maxillary sinus: A case report. Braz. J. Otorhinolaryngol..

[6] Coates G.M. (1937). Cholesteatoma of the frontal sinus. Arch. Otolaryngol..

[7] Thacker E.A. (1950). Epidermoid tumors of the frontal bone, sinus and orbit. Arch. Otolaryngol..

[8] Osborn D.A., Wallace M. (1967). Carcinoma of the frontal sinus associated with epidermoid cholesteatoma. J. Laryngol. Otol..

[9] Calcaterra T.C., Schwartz H.E. (1976). Cholesteatoma of the frontal sinus. Trans. Sect. Otolaryngol. Am. Acad. Ophthalmol. Otolaryngol..

[10] Holt G.R., Holt J.E., Davis W.E. (1977). Late recurrence of a frontal sinus cholesteatoma. Ann. Otol. Rhinol. Laryngol..

[11] Maniglia A.J., Villa L. (1977). Epidermoid carcinoma of the frontal sinus secondary to cholesteatoma. Trans. Sect. Ophthalmol. Am. Acad. Ophthalmol. Otolaryngol..

[12] Campanella R.S., Caldarelli D.D., Friedberg S.A. (1979). Cholesteatoma of the frontal and ethmoid areas. Ann. Otol. Rhinol. Laryngol..

[13] Newman A.N., Colman M., Jayich S.A. (1983). Verrucous carcinoma of the frontal sinus: A case report and review of the literature. J. Surg. Oncol..

[14] Hopp M.L., Montgomery W.W. (1984). Primary and secondary keratomas of the frontal sinus. Laryngoscope.

[15] Hansen S., Sørensen C.H., Stage J., Mouritzen A., Cayé-Thomasen P. (2007). Massive cholesteatoma of the frontal sinus: Case report and review of the literature. Auris Nasus Larynx.

[16] Hammami B., Mnejja M., Chakroun A., Achour I., Chakroun A., Charfeddine I., Ghorbel A. (2010). Cholesteatoma of the frontal sinus. Eur. Ann. Otorhinolaryngol. Head Neck Dis..

[17] Lai J.C., Liu C.K., Chen M.L., Chen M.K. (2010). Removal of frontal sinus keratoma solely via endoscopic sinus surgery. J. Laryngol. Otol..

[18] Zoia C., Pusateri A., Carena P., Matti E., Galioto S., Benazzo M., Gaetani P., Pagella F. (2015). Frontal sinus cholesteatoma with intracranial complication. ANZ J. Surg..

[19] Kurien R., Thomas L., Varghese L., Nair B.R. (2019). Frontal sinus cholesteatoma: A masquerading diagnosis. BMJ Case Rep..

[20] Tejani N., Kshirsagar R., Song B., Liang J. (2020). Evolving treatment of frontal sinus cholesteatoma: A case report. Perm. Med. J..

[21] Gonçalves D.C., Santos T.S., Miranda Silva V.C., Costa H.N.A., Carvalho C.M.F. (2022). Frontal sinus cholesteatoma presenting with intracranial and orbital complications: Diagnosis and treatment. Turk. Arch. Otorhinolaryngol..

[22] Mills C.P., Sycamore E.M. (1958). Cholesteatoma of the maxillary antrum. J. Laryngol. Otol..

[23] Pogorel B.S., Budd E.G. (1965). Cholesteatoma of the maxillary sinus: A case report. Arch. Otolaryngol..

[24] Baxter J.S. (1966). Cholesteatoma of the maxillary antrum. J. Laryngol. Otol..

[25] Das S.K. (1971). Cholesteatoma of maxillary sinus. J. Laryngol. Otol..

[26] Paaske P.B. (1984). Cholesteatoma of the maxillary sinus (a case report). J. Laryngol. Otol..

[27] Sadoff R.S., Pliskin A. (1989). Cholesteatoma (keratoma) of the maxillary sinus: Report of a case. J. Oral Maxillofac. Surg..

[28] Storper I.S., Newman A.N. (1992). Cholesteatoma of the maxillary sinus. Arch. Otolaryngol. Head Neck Surg..

[29] Vaz F., Callanan V., Leighton J., Risdon R.A. (2000). Congenital maxillary sinus cholesteatoma. Int. J. Pediatr. Otorhinolaryngol..

[30] Palacios E., Robertson H. (2005). Cholesteatoma of the maxillary sinus. Ear Nose Throat J..

[31] Viswanatha B., Nayak L.K., Karthik S. (2007). Cholesteatoma of the maxillary sinus. Ear Nose Throat J..

[32] Viswanatha B. (2011). Cholesteatoma of the nose and maxillary and ethmoid sinuses: A rare complication of palatal surgery. Ear Nose Throat J..

[33] Chouhan M., Yadav J.S., Bakshi J., Saikia U.N. (2011). Cholesteatoma of maxillary sinus: Mimicking as sinus tumor. Clin. Rhinol. Int. J..

[34] Burić N., Jovanović G., Tijanić M. (2013). Usefulness of cone-beam CT for presurgical assessment of keratoma (cholesteatoma) of the maxillary sinus. Head Neck.

[35] Sozansky J., Josephson J.S. (2015). Cholesteatoma of the maxillary sinus: A case report and review of the literature. Am. J. Otolaryngol..

[36] Jin H., Shin J.H., Kim K.S. (2016). Cholesteatoma of maxillary sinus: What is the best surgical approach?. J. Craniofac. Surg..

[37] Bourchom W., Jaruchinda P. (2017). Cholesteatoma of the maxillary sinus. Ann. Clin. Case Rep..

[38] Vakalapudi S., Majumdar S., Uppala D. (2021). Cholesteatoma of maxillary sinus simulating neoplasia: A rare case report. Int. J. Appl. Basic Med. Res..

[39] Zhen H., Peng L., Niu D. (2022). A double intranasal approach to treat cholesteatoma of the maxillary sinus: A report of two cases and a literature review. B-ENT.

[40] Cingi E., Cingi C. (1991). Ethmoidal cholesteatoma. Ann. Otol. Rhinol. Laryngol..

[41] Barnett F.C., Barnett J.C. (1992). Massive bifrontal epidermoid tumor. Surg. Neurol..

[42] Chandra R.K., Palmer J.N. (2006). Epidermoids of the paranasal sinuses and beyond: Endoscopic management. Am. J. Rhinol..

[43] Ohta S., Nishizawa S., Ryu H., Yokoyama T., Hinokuma K., Yamaguchi M., Uemura K. (1997). Epidermoid tumor in the sphenoid sinus–case report. Neurol. Med. Chir..

[44] Sani S., Smith A., Leppla D., Ilangovan S., Glick R. (2005). Epidermoid cyst of the sphenoid sinus with extension into the sella turcica presenting as pituitary apoplexy: Case report. Acta Neurochir..

[45] Kanjanawasee D., Chaowanapanja P., Keelawat S., Snidvongs K. (2019). Sphenoid sinus cholesteatoma—Complications and skull base osteomyelitis: Case report and review of literature. Clin. Med. Insights Case Rep..

[46] Çukurova I., Demirhan E., Karaman Y., Yigitbaşı O.G. (2009). Extraordinary pathologic entities within the concha bullosa. Saudi Med. J..

[47] Michaels L. (1988). Origin of congenital cholesteatoma from a normally occurring epidermoid rest in the developing middle ear. Int. J. Pediatr. Otorhinolaryngol..

[48] Yañez-Siller J.C., Wentland C., Bowers K., Litofsky N.S., Rivera A.L. (2022). Squamous cell carcinoma of the temporal bone arising from cholesteatoma: A case report and review of the literature. J. Neurol. Surg. Rep..

[49] Selleck A.M., Desai D., Thorp B.D., Ebert C.S., Zanation A.M. (2016). Management of frontal sinus tumors. Otolaryngol. Clin. N. Am..

[50] Vázquez A., Baredes S., Setzen M., Eloy J.A. (2016). Overview of frontal sinus pathology and management. Otolaryngol. Clin. N. Am..

